# Sensitivity and specificity of plasma disappearance rate of indocyanine green as a prognostic indicator in acute liver failure

**DOI:** 10.1186/1471-230X-9-91

**Published:** 2009-12-03

**Authors:** Uta Merle, Olivia Sieg, Wolfgang Stremmel, Jens Encke, Christoph Eisenbach

**Affiliations:** 1 Department of Gastroenterology, University Hospital Heidelberg, Im Neuenheimer Feld 410, 69120 Heidelberg, Germany; 2 Dept. of Internal Medicine, Johanna-Etienne-Krankenhaus Neuss, Neuss, Germany

## Abstract

**Background:**

In patients presenting with acute liver failure (ALF) prediction of prognosis is vital to determine the need of transplantation. Based on the evidence that plasma disappearance rate of indocyanine green (ICG-PDR) correlates with liver cell function, we evaluated the ability of ICG-PDR measured by pulse dye densitometry to predict outcome in patients with acute liver failure.

**Methods:**

Prospectively markers of hepatocellular injury, synthesis and excretion, including ICG-PDR were measured daily until liver transplantation, death, discharge from intensive care unit, or up to 7 days in 25 patients with acute liver failure. Receiver operating curve (ROC) analysis was performed to assess the value of ICG-PDR to predict outcome in ALF.

**Results:**

The 25 patients analyzed included 18 that recovered spontaneously and 7 that underwent liver transplantation (n = 6) or died (n = 1). Causes of ALF included viral hepatitis (n = 4), toxic liver injury (n = 15), ischemic liver injury (n = 2), and cryptogenic liver failure (n = 4). King's college criteria were fulfilled in 85.7% of patients not recovering spontaneously and in 16.7% of patients recovering spontaneously. The mean ICG-PDR measured on day 1 in patients recovering spontaneously was 12.0 ± 7.8%/min and in patients not recovering spontaneously 4.3 ± 2.0%/min (P = 0.002). By ROC analysis the sensitivity and specificity of an ICG-PDR value ≤ 6.3%/min on study day 1 were 85.7% and 88.9%, respectively, for predicting a non spontaneous outcome in ALF.

**Conclusion:**

ICG-PDR allows early and sensitive bedside assessment of liver dysfunction in ALF. Measurement of ICG-PDR might be helpful in predicting the outcome in acute liver failure.

**Trial registration:**

Clinicaltrials.gov, NCT 00245310

## Background

Acute liver failure (ALF) is a rare condition in which rapid deterioration of liver function occurs in previously healthy individuals. The most prominent causes include drug-induced liver injury, viral or autoimmune hepatitis, Wilson disease, ischemia, or unknown reasons [1]. The most widely accepted definition of ALF includes evidence of coagulopathy, usually an INR ≥ 1.5, and any degree of encephalopathy in a patient without pre-existing liver disease and with an illness of <26 weeks duration [2]. Liver transplantation is the treatment of choice, but because this is only clearly justifiable in patients who would otherwise die, criteria for determining such patients have been established. Prognostic scoring systems still fail to achieve success, given the wide variety of aetiologies that lead to this end stage syndrome. The decision as to whether a patient will recover with conservative management or require transplantation has been the subject of many different reports and case series; however, the King's College Criteria remain the current standard for many clinicians [3].

Indocyanine green (ICG) is a water-soluble, inert anionic compound. After intravenous injection it mainly binds to albumin and β-lipoproteins in the plasma. ICG is not metabolized and is almost completely excreted into the bile without enterohepatic recirculation [4]. Because of these features, ICG has been studied in assessing liver function before hepatectomy, in liver transplantation donors and recipients, in patients with chronic liver failure, and in critically ill patients as a prognostic factor [5-11]. Plasma disappearance rate of ICG (ICG-PDR) is the most commonly used ICG-derived parameter for clinical and experimental assessment of liver function and should physiologically be >18%/min [12]. ICG-PDR depends on ICG uptake by hepatocytes, its excretion into the bile, blood flow-dependent liver metabolism, and energy status. It does not represent the liver blood flow alone. There are various techniques to assess ICG-PDR in vivo. The gold standard relies on serial blood sampling after ICG injection with spectrophotometric analysis of concentration [9,10]. However, this method is invasive, expensive and time-consuming. In contrast, a non-invasive pulsedensitometric method uses a transcutaneous system adapted from pulse oxymetry and a peripheral or central venous access for ICG injection. This method correlates well with the invasive method in hemodynamically stable and mechanically ventilated critically ill patients as well as hemodynamic unstable patients undergoing liver transplantation [13,14].

Using ICG-PDR we measured residual liver function in patients with acute liver failure non-invasively. We evaluated the relationship between ICG-PDR and non-spontaneous recovery, to serve as a diagnostic marker to identify candidates who have to be transplanted. We then assessed the usefulness of this test by examining the correlations between the results of ICG-PDR and other markers of liver function.

## Methods

### Patients

From 01/2006 to 02/2007, 25 consecutive patients who presented at the University Hospital of Heidelberg with acute liver failure were prospectively enrolled in this study after obtaining informed consent. The study protocol was approved by the ethics committee of the University of Heidelberg. King's College Hospital criteria and model of end-stage liver disease score were determined as previously defined [3,15]. The main demographic and clinical features of the patients are summarized in Table 1.

**Table 1:** Demographic and clinical features of patients

Variables	All (n = 25)	Spontanous recovery (n = 18)	No spontanous recovery (n = 7)	P*
Mean age ± SD	42.1 ± 19.0	38.3 ± 19.4	51.7 ± 15.2	0.090
Sex (% male)	24.0	22.2	28.6	0.744
Mean bilirubin [mg/dL ± SD]	10.2 ± 10.7	6.6 ± 6.5	19.2 ± 14.3	0.079
Mean creatinine [mg/dL ± SD]	1.3 ± 2.2	0.7 ± 0.3	2.7 ± 3.9	0.061
Mean INR ± SD	2.4 ± 1.3	1.9 ± 0.4	3.9 ± 1.6	< 0.001
Mean AST [U/L ± SD]	3244.7 ± 3600.9	2903.0 ± 3625.9	4123.4 ± 3655.9	0.333
Mean ALT [U/L ± SD]	2893.9 ± 2282.9	2772.8 ± 2449.6	3205.3 ± 1920.4	0.397
MELD score ± SD	22.9 ± 8.1	18.8 ± 4.8	33.4 ± 3.7	< 0.001
KCH criteria (% fulfilled)	36.0	16.7	85.7	< 0.001
APACHE II	9.1 ± 6.3	7.0 ± 3.9	14.4 ± 8.3	0.042

*Statistical significance of differences between ALF patients with and without spontanous recovery.

Abbreviations: INR, international normalized ratio; MELD, model for end-stage liver disease; KCH, King's College Hospital; and SD, standard deviation.

The clinical management of the patients and the decision of whether to transplant a patient were solely left to the attending physicians discretion blinded to the ICG-PDR measurements. The decision of the attending staff to list a patient for transplantation was based on critical evaluation of performance in clinical scores, the patients general condition and comorbidities as well as careful interpretation of the clinical course.

### Pulse dye densitometry

Pulse dye densitometry was performed using a non-invasive densitometer (LiMON Leberfunktionsmonitor, Pulsion Medical Systems AG, Munich, Germany) by one medical student. The instrument measured arterial ICG concentration based on the principle of differences in absorbance between oxyhaemoglobin and ICG (wavelengths, 905 nm and 805 nm, respectively). This procedure is similar to the concept of pulse oximetry, in which arterial oxygen saturation represents the difference in adsorbance between oxyhaemoglobin and reduced haemoglobin (wavelengths 940 nm and 660 nm, respectively). In principle, the ICG-PDR is determined by monoexponential transformation of the original ICG concentration curve, backward extrapolation to the time point 'zero' (100%), and describing the decay as percentage change per time. Taking blood samples is not necessary.

Directly after inclusion in the study a bolus of 25 mg of ICG (ICG-Pulsion, Pulsion Medical AG, München. Germany) was injected through an intravenous catheter, followed immediately by flushing with physiological saline. Blood ICG concentrations are monitored at every pulse via an optical probe attached to the patient's finger. ICG-PDR means the change of ICG concentration over time (in percent per minute) and reflects the amount of the dye which is eliminated in percent of the initial value. It is automatically calculated by drawing the decay curve of ICG density. After inclusion in the study ICG-PDR was determined daily until death, liver transplantation, or discharge from intensive care unit for up to 7 days. During the study no adverse events attributable to ICG application were noted. Blood samples were taken daily during the study to assess serum concentrations of the following liver function tests and markers for disease severity/liver injury: aspartate aminotransferase (AST), alanine aminotransferase (ALT), bilirubin, creatinine, cholinesterase, albumin, INR.

### Statistical analysis

The Mann-Whitney U test was used to examine differences in laboratory test results, ICG-PDR values, Apache II scores, and MELD scores between 2 groups and the Kruskal Wallis test to examine differences between more than 2 groups. For the measures of diagnostic accuracy 95% confidence intervals are reported. Correlations between ICG and results of other laboratory tests and scores were determined using Pearson's correlation coefficient. P values less than 0.05 were considered statistically significant.

## Results

### Characteristics of the study population and outcome

The study group included 19 women and 6 men with a mean age of 42.1 years (range: 18 - 80 years). Causes of ALF included viral hepatitis (n = 4), toxic liver injury (n = 15), ischemic liver injury (n = 2), and cryptogenic liver failure (n = 4). ALF patients with toxic liver injury included four patients with acetaminophen (AAP) and 11 patients with other drug or Amanita mushroom poisoning (non-acetaminophen, NAAP). The total of 25 patients included 18 that recovered spontaneously and 7 that underwent liver transplantation (n = 6) or died (n = 1). Among the different causes, patient numbers with versus without spontaneous recovery were: viral hepatitis (2/2), AAP (4/0), NAAP (8/3), ischemic (1/1), and cryptogenic liver failure (3/1).

The laboratory data on admission showed that the patients that recovered spontaneously had a significantly lower INR (1.9 ± 0.4 vs. 3.9 ± 1.6, P < 0.001). Bilirubin and creatinine in serum were both higher in patients that did not recover spontaneously, although the difference was not significant (Table 1). The MELD score on admission was significantly higher in the patients that did not recover spontaneously (33.4 ± 3.7 vs. 18.8 ± 4.8, P < 0.001).

King's college criteria were fulfilled in 6 of 7 patients not recovering spontaneously and negative in 15 of 18 patients recovering spontaneously, resulting in a sensitivity of 85.7% and a specificity of 83.3%.

### Measurement of ICG-PDR in ALF patients

**Figure 1:**
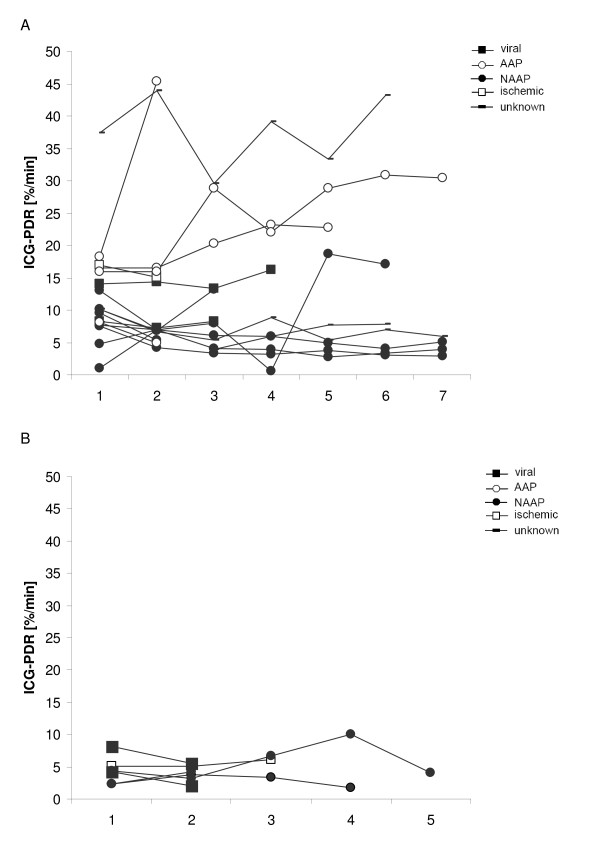
**Time course of indocyanine green plasma disappearance rate (ICG-PDR) in ALF patients with (A) spontaneous recovery (n = 18) and (B) no spontaneous recovery (n = 7)**. ICG-PDR was obtained daily during the study (day 1 to 7 or until liver transplantation, death, or discharge from intensive care unit).

ICG-PDR was measured daily in each patient from admission for seven consecutive days. Measurements were terminated earlier in patients that were liver transplanted, died, or were discharged from intensive care unit. In all of the patients, the first ICG-PDR measurement was made within 24 h after admission to the intensive care unit. The course of ICG-PDR values for each ALF patient is displayed in Figure 1A for patients recovering spontaneously and in Figure 1B for patients that did not recover spontaneously.

**Figure 2:**
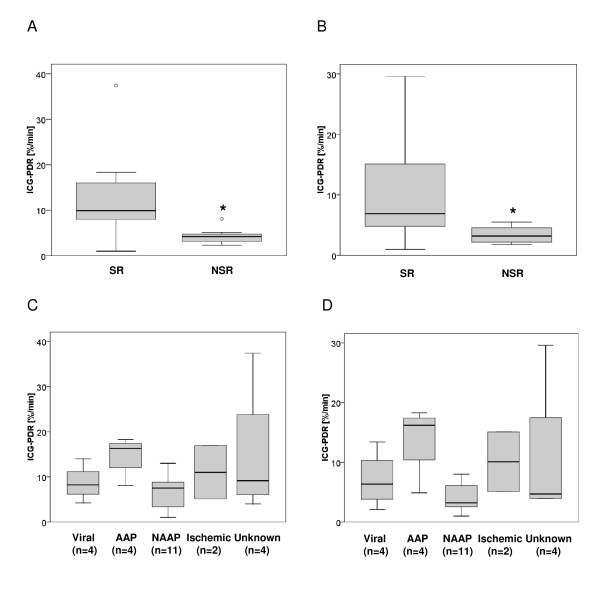
**Indocyanine green plasma disappearance rate (ICG-PDR) in patients with acute liver failure**. Distribution of data presented as a box and whisker plots: 25th percentile, median, 75th percentile, maximum, and outliers (circles). The bold asterisk indicates statistical significance (Mann-Whitney *U *test). (A) ICG-PDR measured on day 1 in patients with ALF that recovered spontaneously (SR) or did not recover spontaneously (NSR). ICG-PDR is significantly lower in patients that did not recover spontaneously than in patients that recovered spontaneously (P = 0.002). (B) Lowest ICG-PDR measured throughout the study (day 1-7) in patients with ALF that recovered spontaneously (SR) or did not recover spontaneously (NSR). ICG-PDR is significantly lower in patients that did not recover spontaneously than in patients that recovered spontaneously (P = 0.018). (C) ICG-PDR measured on day 1 in different causes of ALF including viral hepatitis, acetaminophen (AAP), and non-acetaminophen (NAAP) induced liver injury, ischemic liver injury, and unknown causes. The number of patients in each category is indicated. Using Kruskal-Wallis test, no statistical difference was observed between the different etiological ALF groups. (D) Lowest ICG-PDR measured throughout the study (day 1-7) in different causes of ALF including viral hepatitis, acetaminophen (AAP), and non-acetaminophen (NAAP) induced liver injury, ischemic liver injury, and unknown causes. The number of patients in each category is indicated. Using Kruskal-Wallis test, no statistical difference was observed between the different etiological ALF groups.

The ICG-PDR measured at day 1 was significantly lower in patients not recovering spontaneously than in spontaneously recovering patients (mean, 4.3 ± 2.0%/min; median 13.0; vs. mean, 12.0 ± 7.8%/min; median 4.2, respectively (P = 0.002)) (Figure 2A). The lowest ICG-PDR measured in each individual during the study was also significantly lower in patients not recovering spontaneously than in spontaneously recovering patients (mean, 3.4 ± 1.5%/min; median 6.9; vs. mean, 9.4 ± 7.3%/min, median, 3.2, respectively (P = 0.018)) (Figure 2B). There was no statistically significant difference between the ICG-PDR measured at day 1 and the lowest ICG-PDR measured in each individual patient. When comparing patients' ICG-PDR with respect to the aetiology of ALF no statistically significant differences between the 5 groups could be detected. This was true for ICG-PDR at day 1 and for the lowest ICG-PDR measured in each individual (Figures 2C and 2D).

**Figure 3:**
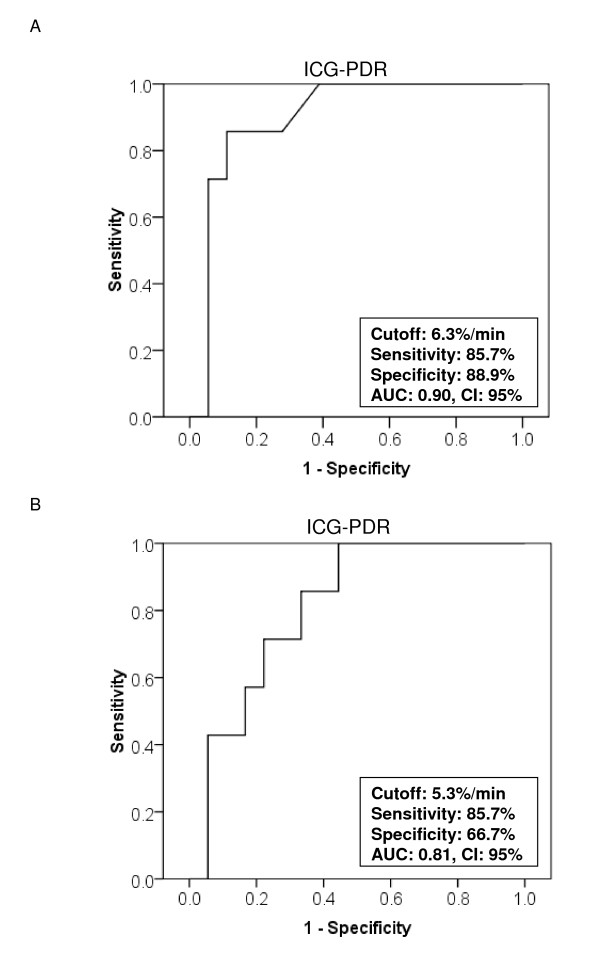
**Predictive discrimination of (A) ICG-PDR measured at day 1 of study and (B) the lowest ICG-PDR measured in each ALF patient during the study as determined by receiver operating characteristics (ROC) plot analysis**. The ROC analysis indicates the ICG-PDR threshold for the best compromise sensitivity/specificity to predict ALF outcome (AUC, area under the curve; CI, confidence interval).

ROC statistics using ICG-PDR measured day 1 (Figure 3A) revealed an AUC of 0.90 (95% confidence interval, 0.77-1.03) and ROC statistics using lowest ICG-PDR measured in each individual ALF patient (Figure 3B) revealed an AUC of 0.81 (95% confidence interval, 0.64-0.98). An ICG-PDR ≤ 6.3%/min measured on day 1 predicted death or transplantation with a sensitivity of 85.7% (95% confidence interval, 42.0%-99.3%) and a specificity of 88.9% (95% confidence interval, 64.0%-98.0%). The lowest ICG-PDR measured in each individual patient at any timepoint = 5.3%/min predicted death or transplantation with a sensitivity of 85.7% (95% confidence interval, 42.0%-99.3%) and a specificity of 66.7% (95% confidence interval, 41.1%-85.6%).

When analyzing correlation of ICG-PDR measured at day 1 with INR, bilirubin, creatinine, AST, ALT, Apache II score, and MELD score, significant correlations were obtained for INR (r = -0.41; P = 0.043), bilirubin (r = -0.53, P = 0.006), and MELD score (r = -0.67; P < 0.001).

## Discussion

Based upon the available data, the current prognostic scoring systems have not consistently demonstrated reliable accuracy in predicting outcome from ALF and the subsequent need for liver transplantation [16]. In this study we prospectively evaluated ICG-PDR in predicting outcome in 25 patients with ALF.

ICG-PDR measured at day 1 (after inclusion in the study) below 6.3%/min had a sensitivity of 85.7% and a specificity of 88.9% in predicting outcome in ALF patients. When analyzing the lowest ICG-PDR values obtained in each individual patient during the study period (day 1 to 7 or until liver transplantation, death, or discharge from intensive care unit, respectively) the sensitivity remained high, but specificity was slightly lower. Our results coincide with a previously published retrospective analysis of ICG-PDR in seven patients presenting with acute liver failure [17]. In this report ICG-PDR values greater than 5%/min were a prognostic factor for hepatic recovery. However, although in our cohort the predictive value of ICG-PDR is promising, caution has to be taken with the interpretation of single ICG-PDR measurement as ICG-PDR may vary from day to day (Figure 1). One could speculate that the observed variation of ICG-PDR values may in part be caused by an - to date speculative - additional extrahepatic ICG elimination or decay. This hypothesis is supported by the previously published observation of ICG clearance even in anhepatic patients [18]. Nevertheless, our results suggest that ICG-PDR measured early during the course of ALF is a valuable prognostic marker for predicting outcome of ALF. One advantage is its prognostic value early in the course of ALF. Thus, measurement of ICG-PDR might help to timely identify those patients with ALF who are likely to benefit from liver transplantation.

To date the most widely used criteria are the King's college criteria [3]. In a recent meta-analysis, Bailey et al. [16] compared different prognostic criteria used to determine the need for liver transplantation and found that all prognostic models presently available lack sensitivity and may miss patients requiring transplantation. In this meta-analysis King's college criteria performed best, with a sensitivity of 69% and a specificity of 92%. In our study sensitivity and specificity of King's college criteria in predicting outcome in ALF patients were 85.7% and 83.3%, and thus in the range of the results of the studies included in the meta-analysis of Bailey et al. [16]. Compared to the results obtained in the meta-analysis of Bailey et al. [16] the prognostic value of ICG-PDR in our recent study is superior to King's college criteria with respect to its sensitivity and equal with respect to its specificity. However, the evaluation of the performance of King's College criteria in our and other studies must be interpreted with care as fulfilment of the criteria has most likely influenced the clinical decision whether to transplant a patient or not. In addition the number of patients analyzed in our present study is limited. Thus, future studies in larger cohorts will have to follow.

Clinical studies in ALF patients are often limited because of heterogeneous aetiologies, the small number of cases, and difficulties in predicting the patient's outcome without transplantation. In our present study predicting the spontaneous outcome of six of the seven patients not recovering spontaneously and receiving liver transplantation can only be assumed fatal. However, as the fatal outcome in these is only an assumption and part may have survived without liver transplantation the calculated ICG-PDR test performances may be inferior to that stated.

In general, ICG removal from blood depends on liver blood flow, parenchymal cellular function and biliary excretion [19,20]. ICG-PDR is a complex measure of both, sinusoidal perfusion as well as hepatic cell membrane function, and thus, reflects a functional reserve of intact hepatocytes which participate in maintained nutritional perfusion [21]. In our study, ICG-PDR allowed early and sensitive detection of spontaneous recovery from ALF, which might be helpful in clinical management. A non-spontaneous recovery from ALF was associated with high ICG retention rates. The advantage of the method used in our study is that ICG-PDR can be measured noninvasively at the bedside by a transcutaneous system even in critically ill patients and results can be obtained within 6-8 minutes [13]. Normal values for ICG-PDR are considered to be over 18%/min. In our study only one patient presenting with ALF had an ICG-PDR in the normal range. ICG is known to be a safe substance, as side effects are very rare (1:40000). Since ICG contains iodine, however, it should not be used in patients with iodine allergy or thyreotoxicosis.

## Conclusion

ICG-PDG is a valuable dynamic liver function tool to predict outcome in patients suffering from ALF early during its course. Because of the possibility of its user-friendly non-invasive bedside assessment, we suggest to use ICG-PDR together with other clinical variables to monitor progress or reversal of liver failure.

## Competing interests

The authors declare that they have no competing interests.

## Authors' contributions

UM performed the statistical analysis and drafted the manuscript. OS carried out ICG measurements. WS helped to draft the manuscript. JE conceived of the study and participated in its design. CE conceived of the study, and participated in its design and coordination and helped to draft the manuscript. All authors read and approved the final manuscript.

## Pre-publication history

The pre-publication history for this paper can be accessed here:

http://www.biomedcentral.com/1471-230X/9/91/prepub

## References

[B1] Ostapowicz G Fontana RJ Schiodt FV Larson A Davern TJ Han SH McCashland TM Shakil AO Hay JE Hynan L Results of a prospective study of acute liver failure at 17 tertiary care centers in the United States Ann Intern Med 2002 137 12 947 954 12484709 10.7326/0003-4819-137-12-200212170-00007

[B2] Polson J Lee WM AASLD position paper: the management of acute liver failure Hepatology 2005 41 5 1179 1197 10.1002/hep.20703 15841455

[B3] O'Grady JG Alexander GJ Hayllar KM Williams R Early indicators of prognosis in fulminant hepatic failure Gastroenterology 1989 97 2 439 445 2490426 10.1016/0016-5085(89)90081-4

[B4] Wu CC Ho WL Yeh DC Huang CR Liu TJ P'Eng FK Hepatic resection of hepatocellular carcinoma in cirrhotic livers: is it unjustified in impaired liver function? Surgery 1996 120 1 34 39 10.1016/S0039-6060(96)80238-8 8693420

[B5] Yamanaka N Okamoto E Oriyama T Fujimoto J Furukawa K Kawamura E Tanaka T Tomoda F A prediction scoring system to select the surgical treatment of liver cancer. Further refinement based on 10 years of use Ann Surg 1994 219 4 342 346 10.1097/00000658-199404000-00003 8161258 PMC1243149

[B6] Midorikawa Y Kubota K Takayama T Toyoda H Ijichi M Torzilli G Mori M Makuuchi M A comparative study of postoperative complications after hepatectomy in patients with and without chronic liver disease Surgery 1999 126 3 484 491 10486600

[B7] Hemming AW Scudamore CH Shackleton CR Pudek M Erb SR Indocyanine green clearance as a predictor of successful hepatic resection in cirrhotic patients Am J Surg 1992 163 5 515 518 10.1016/0002-9610(92)90400-L 1575310

[B8] Okamoto E Kyo A Yamanaka N Tanaka N Kuwata K Prediction of the safe limits of hepatectomy by combined volumetric and functional measurements in patients with impaired hepatic function Surgery 1984 95 5 586 592 6324403

[B9] Plevris JN Jalan R Bzeizi KI Dollinger MM Lee A Garden OJ Hayes PC Indocyanine green clearance reflects reperfusion injury following liver transplantation and is an early predictor of graft function J Hepatol 1999 30 1 142 148 10.1016/S0168-8278(99)80018-X 9927161

[B10] Tsubono T Todo S Jabbour N Mizoe A Warty V Demetris AJ Starzl TE Indocyanine green elimination test in orthotopic liver recipients Hepatology 1996 24 5 1165 1171 10.1002/hep.510240531 8903393 PMC2976580

[B11] Sakka SG Reinhart K Meier-Hellmann A Prognostic value of the indocyanine green plasma disappearance rate in critically ill patients Chest 2002 122 5 1715 1720 10.1378/chest.122.5.1715 12426276

[B12] Leevy CM Smith F Longueville J Paumgartner G Howard MM Indocyanine green clearance as a test for hepatic function. Evaluation by dichromatic ear densitometry Jama 1967 200 3 236 240 10.1001/jama.200.3.236 6071462

[B13] Sakka SG Reinhart K Meier-Hellmann A Comparison of invasive and noninvasive measurements of indocyanine green plasma disappearance rate in critically ill patients with mechanical ventilation and stable hemodynamics Intensive Care Med 2000 26 10 1553 1556 10.1007/s001340000639 11126271

[B14] Faybik P Krenn CG Baker A Lahner D Berlakovich G Steltzer H Hetz H Comparison of invasive and noninvasive measurement of plasma disappearance rate of indocyanine green in patients undergoing liver transplantation: a prospective investigator-blinded study Liver Transpl 2004 10 8 1060 1064 10.1002/lt.20205 15390334

[B15] Wiesner R Edwards E Freeman R Harper A Kim R Kamath P Kremers W Lake J Howard T Merion RM Model for end-stage liver disease (MELD) and allocation of donor livers Gastroenterology 2003 124 1 91 96 10.1053/gast.2003.50016 12512033

[B16] Bailey B Amre DK Gaudreault P Fulminant hepatic failure secondary to acetaminophen poisoning: a systematic review and meta-analysis of prognostic criteria determining the need for liver transplantation Crit Care Med 2003 31 1 299 305 10.1097/00003246-200301000-00048 12545033

[B17] Inderbitzin D Muggli B Ringger A Beldi G Gass M Gloor B Uehlinger D Regli B Reichen J Candinas D Molecular absorbent recirculating system for the treatment of acute liver failure in surgical patients J Gastrointest Surg 2005 9 8 1155 1161 discussion 1161-1152 10.1016/j.gassur.2005.07.026 16269387

[B18] Bruegger L Studer P Schmid SW Pestel G Reichen J Seiler C Candinas D Inderbitzin D Indocyanine green plasma disappearance rate during the anhepatic phase of orthotopic liver transplantation J Gastrointest Surg 2008 12 1 67 72 10.1007/s11605-007-0352-3 17960466

[B19] Paumgartner G The handling of indocyanine green by the liver Schweiz Med Wochenschr 1975 105 17 Suppl 1 30 1135620

[B20] Cherrick GR Stein SW Leevy CM Davidson CS Indocyanine green: observations on its physical properties, plasma decay, and hepatic extraction J Clin Invest 1960 39 592 600 10.1172/JCI104072 13809697 PMC293343

[B21] Caesar J Shaldon S Chiandussi L Guevara L Sherlock S The use of indocyanine green in the measurement of hepatic blood flow and as a test of hepatic function Clin Sci 1961 21 43 57 13689739

